# Why Hypothesis Testers Should Spend Less Time Testing Hypotheses

**DOI:** 10.1177/1745691620966795

**Published:** 2020-12-16

**Authors:** Anne M. Scheel, Leonid Tiokhin, Peder M. Isager, Daniël Lakens

**Affiliations:** Human-Technology Interaction Group, Eindhoven University of Technology

**Keywords:** exploratory research, hypothesis testing, replication crisis

## Abstract

For almost half a century, Paul Meehl educated psychologists about how the mindless use of null-hypothesis significance tests made research on theories in the social sciences basically uninterpretable. In response to the replication crisis, reforms in psychology have focused on formalizing procedures for testing hypotheses. These reforms were necessary and influential. However, as an unexpected consequence, psychological scientists have begun to realize that they may not be ready to test hypotheses. Forcing researchers to prematurely test hypotheses before they have established a sound “derivation chain” between test and theory is counterproductive. Instead, various nonconfirmatory research activities should be used to obtain the inputs necessary to make hypothesis tests informative. Before testing hypotheses, researchers should spend more time forming concepts, developing valid measures, establishing the causal relationships between concepts and the functional form of those relationships, and identifying boundary conditions and auxiliary assumptions. Providing these inputs should be recognized and incentivized as a crucial goal in itself. In this article, we discuss how shifting the focus to nonconfirmatory research can tie together many loose ends of psychology’s reform movement and help us to develop strong, testable theories, as Paul Meehl urged.

A modern student of psychology, wanting to learn how to contribute to the science of human cognition and behavior, is typically presented with the following procedure. First, formulate a hypothesis, ideally one deductively derived from a theory. Second, devise a study to test the hypothesis. Third, collect and analyze data. And fourth, evaluate whether the results support or contradict the theory. The student will learn that doubts about the rigor of this process recently caused our discipline to reexamine practices in the field. Excessive leniency in study design, data collection, and analysis led psychological scientists to be overconfident about many hypotheses that turned out to be false. In response, psychological science as a field tightened the screws on the machinery of confirmatory testing: Predictions should be more specific, designs more powerful, and statistical tests more stringent, leaving less room for error and misrepresentation. Confirmatory testing will be taught as a highly formalized protocol with clear rules, and the student will learn to strictly separate it from the “exploratory” part of the research process. Seemingly well prepared to make a meaningful scientific contribution, the student is released into the big, wide world of psychological science.

But our curriculum has glossed over a crucial step: The student, now a junior researcher, has learned how to operate the hypothesis-testing machinery but not how to feed it with meaningful input. When setting up a hypothesis test, the junior researcher has to specify how their independent and dependent variables will be operationalized, how many participants they will collect, which exclusion criteria they will apply, which statistical method they will use, how to decide whether the hypothesis was corroborated or falsified, and so on. But deciding between these myriad options often feels like guesswork. Looking for advice, they find little more than rules of thumb and received wisdom. Although this helps them to fill in the preregistration form, a feeling of unease remains. Should science not be more principled?

We believe that the junior researcher’s unease signals an important problem. What they experience is a lack of knowledge about the elements that link their test back to the theory from which their hypothesis was derived. By using arbitrary defaults and heuristics to bridge these gaps, the researcher cannot be sure how their test result informs the theory. In this article, we discuss which inputs are necessary for informative tests of hypotheses and provide an overview of the diverse research activities that can provide these inputs.

## The Role of the Hypothetico-Deductive Method in Psychology’s Crisis

The process we taught our hypothetical student above is commonly known as the hypothetico-deductive (HD) method. Hypothetico-deductivism is “the philosophy of science that focuses on designing tests aimed at falsifying the deductive implications of a hypothesis” (Fidler et al., 2018, p. 238). An important modification to the HD method was Popper’s critical rationalism (Popper, 1959): Although empirical data never allow us to infer that a theory is true, theories that survive repeated tests with a high capacity to falsify their predictions are more strongly “corroborated” (Fidler et al., 2018). The HD method is so central to research in many fields that it is often equated with the scientific method. Many scientists invoke Popperian hypothetico-deductivism when describing aspects of their research, and the HD method’s prominent role in textbooks suggests that it shapes scientific discourse in many fields, including psychology (Mulkay & Gilbert, 1981; Riesch, 2008; Rozin, 2009).

The HD method played a key part in psychology’s recent replication crisis (Derksen, 2019). This “crisis of confidence” (Pashler & Wagenmakers, 2012) was based on the insight that psychological scientists’ “approach to collecting, analyzing, and reporting data made it too easy to publish false-positive findings” (Nelson et al., 2018, p. 511). The subsequent reform movement emphasized that psychological scientists (a) were motivated to publish mainly “positive” results that support a tested hypothesis and (b) had “enough leeway built into a study [that] researchers could show just about anything” (Spellman, 2015, p. 887). That is, the crisis was described as hypothetico-deductivism gone awry: Hypotheses were tested, but the tests were weak and their interpretations were warped, resulting in overconfidence and false inferences.

Reforms proposed in reaction to the crisis tried to repair the HD machinery by making methods more rigorous (Spellman, 2015). One influential proposal was to separate confirmatory (hypothesis-testing) and exploratory (hypothesis-generating) research using preregistration (Wagenmakers et al., 2012). Many journals began to offer Registered Reports, a format in which peer review and publication decisions take place before data collection and analysis (Chambers & Tzavella, 2020). Because Registered Reports add peer review and editorial oversight to the preregistration process, they provide an even tighter seal against bias and error inflation. Further proposals urged psychological scientists to specify more precise hypotheses (e.g., by defining a smallest effect size of interest, a region of practical equivalence (ROPE) in Bayesian estimation, or Bayesian priors; Harms & Lakens, 2018) and test them with higher statistical power (Fraley & Vazire, 2014).

The story could have ended here. Psychological scientists used to cut corners when testing hypotheses, new practices and standards were developed in response, and now the discipline moves forward. But in our view, this is not what happened. Rather than just closing a loophole, tightening the screws on hypothesis testing has revealed a deeper problem: The *input* for the testing machinery is missing.

## Are Psychological Scientists Ready to Test Hypotheses?

The reform movement has formalized our hypothesis-testing procedures. Preregistering statistical predictions facilitates Type 1 error control and makes the tests’ capacity to falsify these predictions (“severity”; Mayo, 2018) more transparent. Journals increasingly ask for sample-size justifications based on a priori power analyses to control Type 2 error rates. Further, researchers are increasingly expected to design studies that can provide evidence both for and against the predicted effects (*Comprehensive Results in Social Psychology*, 2020) and to specify the conditions to which they expect findings to generalize (Simons et al., 2017).

In practice, however, researchers have substantial difficulties incorporating these recommendations in their research, and even preregistration’s most ardent proponents acknowledge that “Preregistration Is Hard” (Nosek et al., 2019). Although it is tempting to assume that these difficulties can be resolved by better training and that “the field collectively needs to go through a learning phase” (Claesen et al., 2019, pp. 20–21), we doubt that inexperience is the real problem. Instead, we see several symptoms of problems that require more than practice to solve.

First, even preregistered hypothesis tests are rarely specified in a way that eliminates flexibility in data analysis, with unambiguous criteria for concluding that a prediction is corroborated or falsified (Lakens & DeBruine, 2021; Bakker et al., 2018). The insight that psychologists struggle to define their hypotheses will not surprise those who have criticized psychologists’ practice of null-hypothesis significance testing (NHST) as “the null ritual” (Gigerenzer, 2004). Researchers using NHST typically do not specify their research hypothesis more precisely than as the complement of *H*_0_. NHST can only reject the null—it cannot accept it. Psychological scientists have not developed methods for specifying the alternative hypothesis in sufficient detail to make it statistically falsifiable (Meehl, 1967; Morey & Lakens, 2016). This problem is not solved with mere practice—forcing researchers to specify what would falsify their hypotheses when they have no theoretical basis for doing so can lead to testing against arbitrary values (Kruschke, 2018) and runs the risk of replacing one mindless ritual with another.

Second, if psychological scientists were ready to use formal hypothesis tests, then arduous parts of the preregistration process (e.g., justifying the sample size on the basis of the predicted effect size) should be straightforward: Just fill in the numbers. Yet it has been our experience^1^ that even highly motivated researchers cannot define their predictions in statistical terms because they lack knowledge about the strength of their manipulations and the variance of their measures. Instead, power analyses, smallest effect sizes of interest, and Bayesian priors are predominantly based on norms such as “a medium effect size (*d* = 0.5)” or the default settings of researchers’ statistical software (van de Schoot et al., 2017).

Third, if the Reproducibility Project: Psychology (Open Science Collaboration, 2015) taught us one thing about the state of the field, it is that psychologists have difficulty agreeing on whether findings have been successfully replicated (Maxwell et al., 2015). This problem is also reflected in ongoing debates about “hidden moderators” in which failed replications have been dismissed on the grounds that methodological details were varied, although the original theory did not specify the importance of these details (Simons et al., 2017). A striking feature of such replication debates in psychology is that different parties struggle to agree on the basic content of theories. This problem seems difficult to overcome even when researchers make a concerted effort to reconcile their disagreements (Coles et al., in press), suggesting that theoretical models are not specified clearly enough for adversaries to see where their assumptions diverge.

The claim that many psychological theories are critically immature has been leveled against the field so often that psychological scientists may well have grown tired of it (e.g., Fiedler, 2004; Gigerenzer, 1998; Meehl, 1967, 1978, 1990; Muthukrishna & Henrich, 2019). What is new is that efforts to formalize hypothesis tests have led researchers to directly experience the repercussions of testing immature theories: Tightening the screws on the testing machinery has had the unexpected effect of making psychological scientists aware that they may not be ready to test hypotheses. For example, *Nature Human Behaviour* (2020) requires authors of Registered Reports to plan frequentist analyses with 95% power for “the lowest available or meaningful estimate of the effect size or, when using Bayes factors, to “indicate what distribution will be used to represent the predictions of the theory and how its parameters will be specified.” As researchers have started to justify such statistical choices, they have been forced to confront bigger questions (e.g., about measurement, auxiliary assumptions, and theoretical predictions) that they often do not know how to answer.

In this article, we argue that by focusing primarily on *confirmatory* research and jumping straight to the hypothesis test, psychologists too often neglect the groundwork that is necessary to ensure a sound link between the test and the tested theory. Moving from a theoretical framework to a statistical test can be seen as a sequence of specifications based on deductive logic (e.g., deriving a testable model from a theory) and auxiliary assumptions (e.g., deciding how to measure the dependent variable). Meehl (1990) termed this the “derivation chain,” a conjunction of theoretical and auxiliary premises that are necessary to predict observable outcomes. The statistical prediction at the end of a derivation chain is highly specific. Without paying sufficient attention to the elements that link this prediction to the theory, a hypothesis test has unknown validity. As Meehl (1990) put it, “To the extent that the derivation chain from the theory and its auxiliaries to the predicted factual relation is loose, a falsified prediction cannot constitute a strict, strong, definitive falsifier of the substantive theory” (p. 200).

## The Inputs to Informative Hypothesis Tests

What elements are needed for a strong derivation chain? In his classic book *Theory Building*, Dubin (1969) distinguished (a) concept formation, (b) developing measures, (c) establishing relationships between concepts, (d) specifying boundary conditions and auxiliary assumptions, and (e) deriving statistical predictions as necessary steps before testing hypotheses. We briefly summarize each of these steps below and explain why skipping any one of them makes a hypothesis test less informative.

### Concept formation

Translating theoretical predictions into observable outcomes requires that we know what we want to observe. What do we mean by screen time, intrinsic motivation, or depression? Concept formation is the process of defining the building blocks of theories (e.g., Hempel, 1966) and specifying their attributes. Two criteria for good concepts are coherence and differentiation (Gerring, 1999): Concepts need to describe a class of entities with shared attributes and differentiate this class from other concepts. When concepts are not coherent, we risk “conceptual stretching,” wherein a concept does not fit the new cases for which it is used. For example, social psychology borrowed the concept of *priming* from cognitive psychology to explain effects that were argued to last for months, even though priming effects in cognitive psychology lasted only seconds. Problems with a lack of differentiation have been noted regarding the concept *grit*, which may be redundant given its high correlation with conscientiousness (Credé et al., 2017). Without sufficiently defined concepts, we cannot know whether our measures adequately capture them, and the meaning of our test results will remain unclear.

### Measurement

To empirically examine concepts, we need to specify how they will be measured and understand what these measures mean. For example, researchers might assume that different measures are equivalent (e.g., using stated preferences vs. behavioral tasks to measure risk preference; Frey et al., 2017) without realizing that they capture different constructs. Despite the importance of reliable and valid measures, measurement practices in psychology are suboptimal (Borsboom, 2006). Scales are used without evidence of their validity or are simply created on the fly (Flake et al., 2017). Further, measures with low reliability compromise the inferences drawn from hypothesis tests because noise factors obscure causal effects on the dependent variable (Loken & Gelman, 2017; Shadish et al., 2001). Low validity and reliability reduce the extent to which hypothesis tests inform a theory: A positive finding does not support a theory if we manipulated the wrong thing, and a negative finding does not contradict a theory if the dependent variable did not capture the construct of interest. In practice, developing measures often plays out as an iterative back and forth with concept formation, as (for example) problems with a measure’s construct validity can lead to further refinement of the concept (de Groot, 1969).

### Relationships between concepts

Once concepts are sufficiently defined, we need to specify a causal model of how they relate to one another. For example, how exactly should reducing adolescents’ screen time affect their well-being? Psychologists frequently use “box-and-arrow” models without formalizing the implied causal structure, the mathematical functions that relate concepts, or which observations would support and falsify the model (Hernán & Robins, 2020; Pearl, 2009). Should *Y* change if we intervene on *X*? Will *X* and *Y* be statistically independent if we control for *Z*? Failing to consider predictions implied by a causal model can lead to invalid inferences in the presence of selection bias, confounding, and other violations of causal identifiability conditions (Hernán & Robins, 2020). Put simply, if we do not know which effects a causal model predicts, we cannot know whether the model is falsified or corroborated after testing a particular effect.

Without sufficiently defined concepts and information about their causal relations, we lack information about a theory’s *content*: Its scope is unclear, its assumptions are not specified, and its predictions are vague. As a consequence, individuals may interpret the theory in different ways, disagree about its predictions, or test its implications in different conditions. This can result in perpetual disagreement and inconclusive debates (Loehle, 1987).

### Boundary conditions

A good theory is clear about its boundary conditions (i.e., the regions of the parameter space in which the theory applies). Failing to observe the theory’s predictions in those conditions leads to reduced confidence in the theory. A lack of precision and transparency about boundary conditions makes it difficult to interpret empirical discrepancies (e.g., why an effect was not successfully replicated; Simons et al., 2017) and can lead to degenerative research lines (in which modifications are made to accommodate failed predictions without improving the theory’s predictive success; Lakatos, 1978). Without knowing the conditions in which a phenomenon should occur, it is not possible to evaluate the extent to which observing the phenomenon provides evidence for or against a theory.

### Auxiliary assumptions

To test predictions derived from a theory, we rely on additional auxiliary theories or assumptions (Meehl, 1978, 1990). Auxiliaries are claims not directly derived from our theory but that are necessary for translating statements about theoretical constructs into statements about observables. For example, to experimentally test whether feeling socially excluded increases sensitivity to physical pain, we need to assume that our manipulation induces feelings of social exclusion and does not influence pain sensitivity in unintended ways, that group assignment is random, that participants complete the task as intended, and so on. When the validity of auxiliaries is unknown, hypothesis tests are less informative because negative results may result from faults in the auxiliaries instead of faults in the substantive theory (Meehl, 1990).

### Statistical predictions

The inferences we can draw from statistical tests depend on the specificity of the theoretical predictions and on the capacity of tests to falsify them (Mayo, 2018). Thus, when preregistering confirmatory analyses, researchers should specify which findings would support and falsify their hypotheses and indicate the test’s capacity to provide informative results (e.g., statistical power, sensitivity). In practice, researchers must make many decisions, including which sample size to use, which effect sizes are theoretically predicted or practically meaningful, or how to quantify their prior beliefs. If researchers lack a principled way to make these decisions, they may rely on arbitrary default values, and subsequent test results will be arbitrary in return.

## Research Activities to Strengthen the Derivation Chain

All of these inputs determine the strength of the HD derivation chain and the inferences that we can draw from a hypothesis test. Until now, psychology’s reform movement has focused primarily on the final element of the derivation chain: statistical predictions and inferences. However, if researchers struggle with this final part, perhaps the true problem lies further upstream. That is, we may be missing crucial knowledge about auxiliaries, boundary conditions, causal relationships, measures, or concepts. Thus, instead of risking a premature leap from a theoretical idea to a statistical prediction, we may want to ask ourselves: Are we ready to test a hypothesis or would we be better off strengthening the weakest parts of the derivation chain first?

Strengthening the derivation chain requires research activities that are distinct from the final confirmatory test of a prediction. This groundwork constitutes a wide range of nonconfirmatory activities. Some of these activities overlap with theory development (e.g., translating verbal theories into formal models) and psychometric work (e.g., validating a measurement instrument), two areas for which comprehensive advice already exists (e.g., Borsboom et al., 2020; Fried & Flake, 2018), but others are distinct and have received less attention thus far (e.g., exploring boundary conditions, establishing auxiliary assumptions). Below we describe several types of currently underappreciated nonconfirmatory research activities that hypothesis testers can use to strengthen their derivation chains.

### Descriptive and naturalistic observation

Research that is “merely” descriptive is often considered less valuable despite being crucial for forming concepts, developing measures, and establishing phenomena that need explaining (Dubin, 1969; Gerring, 2012a; Rai & Fiske, 2010; Rozin, 2001). Descriptive research answers *what* questions, not *why* questions. Gerring (2012a) outlines various types of descriptive activities, including describing particular accounts, measuring variation across a single dimension, describing associations, grouping entities into a single category, or creating a typology. In research on mental disorders, naturalistic observation of patients’ symptoms often fuels debates about how specific mental disorders should be defined and measured and inspires new models for how these disorders are generated and maintained (e.g., Robinaugh et al., 2019). As an example, Fried and Nesse (2015) used a vast array of observational research on symptoms of depression to show that different symptoms interact in complex but reliable ways that are not captured by the sum-score estimation of major depressive disorder.

### A priori evaluation of theory plausibility

Before testing a theoretically derived hypothesis, it is useful to evaluate the theory’s logical coherence, scope, and plausibility. One approach is to formalize hypotheses via mathematical or computational modeling (Lewandowsky & Farrell, 2010; Smaldino, 2017). Formalization makes theories more transparent and testable by specifying all assumptions, concepts and their relations, and boundary conditions. For example, when Zahavi (1975) proposed the idea that the costliness of signals ensures their reliability (i.e., the handicap principle), many biologists found the idea implausible. Because the idea was specified in natural language, its scope and assumptions were unclear, and initial attempts to formalize it did not produce the predictions Zahavi claimed. After a decade of modeling attempts, a subset of models demonstrated the conditions in which the handicap principle was logically coherent (e.g., condition dependence; differentially costly signals). Only then did researchers empirically test the theory in those conditions (for a review, see Grose, 2011). Without formalization, the theory might have been rejected outright, and the conditions in which it was logically coherent might not have been discovered (for similar issues with prominent verbal theories in social psychology, see Harris, 1976).

Another approach underused in psychology is to assess whether a theory is consistent with principles from existing, highly corroborated theories. For example, terror-management theory (TMT) assumes that humans have an instinct for self-preservation that led to the evolution of an incapacitating fear of death with which humans cope via an anxiety-reducing “terror-management” system (Greenberg et al., 1986). However, some scholars have pointed out that TMT’s assumptions appear to contradict basic tenets of evolutionary theory (Kirkpatrick & Navarrete, 2006). For example, natural selection favors strategies that maximize inclusive fitness (Hamilton, 1964), which is often not accomplished by self-preservation (e.g., people investing less in their future health when extrinsic mortality risks are high; Nettle, 2010). As a result, the assumption that a general survival instinct could evolve has low a priori plausibility. The point is not that a new theory needs to be consistent with every existing theory but rather that some existing theories have been so highly corroborated that they provide informative priors about the verisimilitude of newer theories.

### Parameter-range exploration

Mature theories precisely specify boundary conditions. One way to explore boundary conditions is to move beyond well-studied conditions by traversing a single dimension to determine whether a phenomenon or theory generalizes to the edges of that dimension (i.e., *inside-out exploration*; Busse et al., 2016). Ethologist Nikolaas Tinbergen (1951) discovered the phenomenon of “supernormal stimuli” (i.e., stimuli eliciting stronger behavioral responses than stimuli to which animals evolved to respond) by exploring responses to stimuli exaggerated along single dimensions. For example, by creating unnaturally large eggs, Tinbergen found that female birds had strong preferences for taking care of larger eggs, even when the egg size was far outside its natural range of variation.

A complementary approach involves exploring regions of parameter space in which researchers suspect that a theory might not apply (i.e., *outside-in exploration*; Busse et al., 2016). This is often the motivation for cross-cultural studies in non-WEIRD (Western, educated, industrialized, rich, democratic) populations (e.g., Henrich et al., 2005). For example, Gurven et al. (2013) explored the fit of the five-factor model of personality among the Tsimane, a Bolivian forager-horticulturalist group. The authors found that Tsimane personality variation was better explained by two principal factors, not five, which inspired new theoretical models to explain why the covariance structure among human personality characteristics varies across populations (Smaldino et al., 2019).

Another goal of exploring parameter ranges is to provide information about the functional form of relationships between concepts. In medicine, researchers examine dose-response curves to determine recommended dietary allowances, upper and lower bounds of “healthy” nutrient doses, and tolerable upper-intake levels (e.g., Zittermann, 2014). Establishing manipulation-strength curves by manipulating a variable across a range is more informative than manipulating just two levels (Meehl, 1990). For example, in social-discounting paradigms, participants decide whether to sacrifice some amount of a resource to provide it to other individuals at varying social distances (e.g., the first, fifth, and 20th closest person to you). Using this paradigm, researchers have established that the functional form of the relationship between social distance and willingness to sacrifice is hyperbolic (Jones & Rachlin, 2006; but for issues with generalizability, see Tiokhin et al., 2019). Establishing functional form can inspire deeper questions about phenomena (e.g., why did humans evolve to discount hyperbolically as opposed to linearly?) and reveal connections to phenomena in other domains (e.g., hyperbolic discounting of future rewards; Jones & Rachlin, 2006).

### Exploratory experimentation

Although scientists often think of experiments in the context of confirmation, philosophers of science have emphasized the role of exploratory experiments in theory development (Franklin, 2005; Steinle, 1997, 2002). In exploratory experiments, researchers vary a large number of parameters without a priori predictions of their effects (although some prior knowledge of plausible parameters is necessary), look for stable empirical patterns, and infer rules from these patterns. Exploratory experimentation is widely used in psychophysics to establish law-like relationships (for a discussion of this method in research on face perception, see Jack & Schyns, 2017). In the biological and pharmaceutical sciences, high-throughput experiments were a revolutionary development and are now used to identify the effects of millions of genes, antibodies, and other chemical compounds on biomolecular pathways via “brute-force” experimentation (Mennen et al., 2019; Subramanian et al., 2017).

Steinle (2002) discusses the vital role of exploratory experiments for concept formation in the history of research on electricity. In the early 18th century, the field had generated many interesting but seemingly contradictory effects and lacked a coherent theoretical framework to explain them. In a series of exploratory experiments, Charles Dufay documented which materials could be electrified, what factors influenced the extent of electrification, and how the distance between objects affected their attraction or repulsion. Dufay eventually developed the hypothesis that there were two types of electricity (not one) and that bodies electrified with the same type of electricity repelled one another and vice versa.

### Feasibility and pilot studies

Feasibility and pilot studies are small-scale tests of whether studies work as intended. In medical science, feasibility studies are used to assess recruitment and retention rates, adherence to procedures, rates of unusable responses, and the reliability and validity of measures and to estimate the standard deviation of dependent measures (Eldridge et al., 2016; Lancaster, 2015). Feasibility and pilot studies also provide a way of discovering and examining auxiliary assumptions. For example, when Hruschka et al. (2018) piloted a prototypical social-discounting protocol in rural Bangladesh, they discovered that the protocol confused participants because it relied on auxiliary assumptions about how they would understand and respond to the task (e.g., that moving left to right on a Likert-type scale is a natural way of representing magnitude). Thus, pilot studies are crucial for minimizing the risk that untested auxiliaries and “manipulation-check neglect” (Fiedler, 2018, p. 435) render a study uninformative.

## Strengthening the Derivation Chain in Practice

We use the ongoing research program on *kama muta* to illustrate how nonconfirmatory research activities such as the ones described above can be used to lay the foundation for informative hypothesis tests. *Kama muta* is posited as the sudden experience of a distinct emotion that is characterized in English as being “moved,” “touched,” or having a “heart-warming experience.” The *kama muta* research program is led by an interdisciplinary collaboration, the Kama Muta Lab (KML; see https://kamamutalab.org). Our description draws on several KML publications as well as personal communication with KML founders Alan Fiske, Beate Seibt, and Thomas Schubert.

In the beginning of the research program, the KML invested substantially in concept formation. Such work has relied on a wide range of research activities and sources of evidence, including “ethnological and historical materials, ancient and more recent texts, participant-observation miniethnographies focused on key practices, interviews, diary self-reports Internet blogs and videos, and experiments using self-report responses to controlled stimuli” (Fiske et al., 2017, p. 92). These activities allowed the KML to identify the situational deter-minants of *kama muta* (e.g., witnessing extraordinary acts of kindness, hearing the national anthem, reuniting with an old friend) and its associated bodily sensations (e.g., tearing up, feeling warm in the chest, getting goosebumps). The KML also documented verbal terms for feeling *kama muta* in different languages and cultural practices that evoke *kama muta* (e.g., proscribed weeping at reunions, peace ceremonies, and funerals, which people report as overwhelmingly positive experiences).

Refining the initial concept allowed the KML to create measurement items and compile stimuli (e.g., videos) to invoke the emotion. This made it possible to develop a full scale (KAMMUS Two; Zickfeld et al., 2019), which was validated using cross-cultural self-report data from 19 nations. Whenever the KML found that an item could not be meaningfully translated into a language, the item was removed from all versions of the scale, thus leading to further conceptual refinement.

The causal model of *kama muta*—its proximal causes and consequences as well as its evolved function—was inspired by relational models theory (Fiske, 2004). The KML developed the working hypothesis that *kama muta* arises when “communal sharing relationships (CSRs) suddenly intensify” (Fiske et al., 2019, p. 74) and that it “evokes adaptive motives to devote and commit to the communal sharing relationships that are fundamental to social life” (p. 74). Communal sharing relationships are relationships in which people feel close, equivalent, and that they share a common essence. Knowing how to measure and induce *kama muta* allowed the KML to study the emotion’s structure and its connection with communal sharing in controlled settings. In a time-series analysis of participants’ experiences while watching videos that induced *kama muta*, the KML documented a strong temporal connection between feeling moved, perceived closeness between the video protagonists, and expert ratings of communal sharing (Schubert et al., 2018). However, there were other situations in which people appeared to experience *kama muta* without the intensification of a communal sharing relationship (e.g., performing and listening to certain types of music without the physical presence of others; Fiske et al., 2017). The KML subsequently revised their causal model to posit that *kama muta* was evoked by situations in which communal sharing relationships suddenly became salient (e.g., being reminded of one’s connection to others).

Refining the causal model of *kama muta* required a better understanding of its boundary conditions. Using outside-in exploration (exploring regions of parameter space in which researchers suspect that a phenomenon might not apply), the KML found that participants still felt *kama muta* when the protagonists in stimulus videos had poor reputations (e.g., criminals). This result was surprising given the KML’s working model of *kama muta*’s adaptive function. Another study found that stimuli that were seemingly unrelated to communal sharing relationships (e.g., cute animals) could evoke mild forms of *kama muta* (Steinnes, 2017). Experimentally varying different aspects of stimulus materials thus showed that the boundary conditions of *kama muta* might be broader than previously expected.

Although the *kama muta* research program is still ongoing, the rich existing body of work provides a solid foundation for future research. As an example for how a confirmatory test could be built on this foundation, consider the KML’s hypothesis that a sudden increase in the perceived salience of a communal sharing relationship (rather than experiencing or witnessing an intensification) is enough to trigger the emotion. What would be needed for an informative test of this hypothesis? First, the concepts of “*kama muta*” and “communal sharing relationship” are reasonably well defined, but the meaning of “increased perceived salience” may need further development. Second, although the KAMMUS Two provides a valid measure of *kama muta*, the validity of the current operationalization of communal sharing relationships—a scale measuring “closeness” (Seibt et al., 2018)—may require further investigation. Additional work is also needed to reliably manipulate the onset and magnitude of perceived communal sharing salience (this is the point at which the KML’s inquiry has currently stalled). Third, a causal model is needed to specify the hypothesized relationship between the concepts, as well as relevant third variables that might affect this relationship and the way it can be tested in the lab. Fourth, auxiliary assumptions (needed to translate the test of this model into the lab environment) must be spelled out and examined. Some are already known (e.g., the assumption that the KAMMUS Two reliably measures *kama muta* if the questionnaire is administered and analyzed in a particular way), but others will need to be tested in additional pilot studies (e.g., the assumption that participants process the stimuli in all trials as expected). Finally, with the elements of the derivation chain in place, we would then be ready to translate our hypothesis into a statistical prediction. The effort we invest in the derivation chain pays off as a highly informative test because we know precisely how its outcome is linked to the theoretical premises from which we started.

## Discussion

By tightening the screws on the HD machinery and incentivizing rigorous confirmatory research, psychology’s reform movement may have inadvertently exacerbated the notion of nonconfirmatory research as a “second-class citizen” (Klahr & Simon, 1999, p. 526). We use the term *nonconfirmatory* rather than *exploratory* because we believe the confirmatory-exploratory distinction to be a false dichotomy. Many researchers seem to see exploration as a “chancy” or “mysterious” process (Kerr, 1998, p. 202) with the sole purpose of inspiring new research lines. However, as we hope to have shown in this article, the groundwork that precedes informative confirmatory tests consists of more than being visited by the muse. The research activities we describe above have a clear function: to strengthen the elements of the derivation chain. Because these activities provide researchers with essential knowledge about descriptive phenomena, the content of theories, and auxiliary assumptions, they should form the knowledge base of our discipline instead of being treated as an afterthought to confirmatory research. How, then, can we give such work its rightful place in the literature?

In an effort “to support and promote open-ended, open science, providing a high-status specialized format for its publication” (McIntosh, 2017, p. A2), *Cortex*—the journal that first introduced Registered Reports in 2013—recently launched the new, complementary format Exploratory Reports. However, the number of fitting Exploratory Report submissions has been wanting. One reason may be that an open-ended nonconfirmatory format provides little guidance about how to conduct meaningful research that does not involve hypothesis testing or how to evaluate the scientific value of such work. As a way forward, we suggest that researchers consider which element of their derivation chain is the weakest, such that strengthening it would have the largest effect on the extent to which an eventual hypothesis test can inform a theory.

The concepts of interest should take into account established usage of terms, have a specified domain, be used with consistency, describe referents that share many attributes, be clearly differentiated from other concepts, have theoretical utility, and be operationalizable (Gerring, 2012b). Measures and manipulations of these concepts should be reliable and valid for the population and context of interest (Shadish et al., 2001). The hypothesized causal relationships between target variables should be formalized and take relevant third variables into account, allowing others to judge whether the predicted effect is causally identified (e.g., Rohrer, 2018). Boundary conditions should clearly specify where and when a theory is and is not assumed to hold. Finally, all known auxiliary assumptions should be made explicit and supported by independent studies and/or tested in the form of positive and negative controls.

In practice, judging the quality of these inputs will depend on the specifics of a research area and require an open discourse within the research community. Beyond agreeing on quality standards for the elements of the derivation chain, a remaining challenge will be to ensure that research activities to strengthen these elements do not fall prey to publication bias. Just like confirmatory research, nonconfirmatory research should be transparent and reproducible. Subfields of psychology and neighboring disciplines in which nonconfirmatory research activities are common practice have already begun to tackle these issues (see, e.g., Crüwell et al., 2019; Jacobs, 2020; Moravcsik, 2014). Drawing on existing expertise in these fields, exchanging resources, and starting broader discussions about underused methods may help us overcome our unhealthy fixation on hypothesis tests.

Mainstream psychology rightly prizes HD testing as a powerful tool for drawing inferences about the world. But as long as we do not invest in nonconfirmatory research to supply the inputs to the HD testing machinery, we can fine-tune the motor all we like: The results it spits out will not be informative because the derivation chain linking them back to our theory is broken. Therefore, researchers who want to advance psychological science through hypothesis tests should spend less time testing hypotheses.

## Transparency

*Action Editors:* Travis Proulx and Richard Morey

*Advisory Editor:* Richard Lucas

*Editor:* Laura A. King

*Author Contributions*

A. M. Scheel developed the idea for the manuscript and was responsible for the final structure. All of the authors contributed substantially to the conception of the work, drafted and revised it, approved the final version for submission, and agree to be accountable for all aspects of the work.

## Notes

1. As members of the ethical review board (ERB) at their department, A. M. Scheel and D. Lakens have evaluated several hundred ERB submissions that are required to include a sample-size justification.
